# Book Club at a medical school in the Sertão Region of Rio Grande do Norte State, Brazil

**DOI:** 10.5195/jmla.2026.2177

**Published:** 2026-04-01

**Authors:** Amanda Lima Sampaio, Álida Silva, Yasmin Limão, Lehi Bezerra, Janine Braz

**Affiliations:** 1 amanda.sampaio.096@ufrn.edu.br, Escola Multicampi de Ciências Médicas do RN and Undergraduate Student, Universidade Federal do Rio Grande do Norte, Brazil; 2 alidaandrielly13@gmail.com, Escola Multicampi de Ciências Médicas do RN and Undergraduate Student, Universidade Federal do Rio Grande do Norte, Brazil; 3 ymaria050@gmail.com, Escola Multicampi de Ciências Médicas do RN and Undergraduate Student, Universidade Federal do Rio Grande do Norte, Brazil; 4 lehi.aguiar@ufrn.br, Escola Multicampi de Ciências Médicas do RN and Librarian, Universidade Federal do Rio Grande do Norte, Brazil; 5 janine.braz@ufrn.br, Escola Multicampi de Ciências Médicas do RN and Adjunct Professor, Universidade Federal do Rio Grande do Norte, Brazil

**Keywords:** Medical Education, Book Club, Humanistic Medicine, Literature in medicine

## Abstract

**Background:**

The integration of literature and the humanities into medical education offers numerous benefits for both students and the broader community. Engaging with literary texts encourages the development of empathy, critical thinking, emotional awareness, and communication skills, all essential for a more humanistic and socially responsive medical practice. Additionally, initiatives that bring together students and local residents through shared cultural experiences help strengthen the university’s connection with the surrounding community and promote mutual understanding. The Book club was created at the medical school in Caicó (RN, Brazil), as a space to explore diverse literary works beyond the health sciences.

**Case Presentation:**

The Book Club was approved as an extension event and is promoted in Escola Multicampi de Ciências Médicas do Rio Grande do Norte (EMCM) by the Pro-Rectory of Extension from Universidade Federal do Rio Grande do Norte (UFRN) situated in Caicó city (RN, Brazil). It has a multidisciplinary team of students, teachers and technical staff. Six literary works were selected based on the alignment with the club’s educational goals, particularly the potential to stimulate critical reflection on themes such as grief, racism, gender, and social justice. Registration took place through the Integrated Academic Activities Management System, and the monthly meetings were held in the Medical School auditorium. Participants received pedagogical support via social networks, club app, reading planner and newsletter.

**Conclusions:**

With 22 participants, including 8 community members and 14 medical students, the Book Club explored perspectives such as gender, racism, grief, trauma and colonialism. The predominance of medical students highlights academic interest and institutional support in extension projects, while low community adherence points to the need for new engagement strategies. The analysis of followers on Instagram reveals a significant participation of the local population, especially among women, but also highlights a gap in the presence of men and adolescents. This demonstrates the importance of a more inclusive and diverse approach to attracting different audiences. The Book Club at the public Medical School, by stimulating cognitive and human skills through literature, enriches academic training and strengthens the connection between academia and the community.

## BACKGROUND

Medical training has traditionally followed a predominantly scientific curriculum; however, as the concept of health has evolved to include psychological and social dimensions, the scope of medical practice has also needed to expand [1]. These changes in practice have gained strength in Brazil from the changes in the National Curriculum Guidelines (DCN). In 2001, the DCN first proposed the human and social sciences as core elements within medical curriculum, a position which was subsequently strengthened in 2014 [2].

Humanities education for medical students has been found to be have many benefits [3]. This integration helps medical students develop greater sensitivity and a more comprehensive understanding of human experiences in healthcare [4]. It also fosters essential skills beyond technical knowledge, such as empathy, openness to uncertainty, and more nuanced clinical judgement [5]. Beyond improving care, these skills can also support mental health of students, an increasingly important concern, as emotional exhaustion and career-related discouragement are commonly reported throughout medical training. [6]. Humanities can be introduced through extracurricular activities that reflect the diverse interests contributing to medical education [7] and practice. Reading and reflecting on literature and poetry, for instance, can enhance a doctor’s professional effectiveness by nurturing cognitive and emotional abilities.

Literary narratives, as both artistic expressions and sources of knowledge, play a role in the educational and professional growth of students. Such works can offer a foundation for discussion and reflection, with meanings that resonate differently based on each reader’s perspective and lived experience. In a medical context, an individual patient's narrative about their illness process can often reveal more information and truths than solely scientific approaches [8].

To insert narratives in an academic context, reading clubs are a pedagogical strategy [9,10]. These collective spaces for interaction and dialogue about literary works create an environment conducive to the development of skills that enrich the student's experience with the medical curriculum. In addition, reading clubs promote the development of fundamental skills for a humanized professional practice that is sensitive to the health needs of the population [9]. Empathy is developed by allowing readers to put themselves in the shoes of diverse characters, understanding and feeling their emotions. Communication (verbal and written) is improved through discussions [9].

The benefits extend beyond students and physicians to the broader community, promoting critical thinking, reflection, and literacy [11]. Such initiatives also serve as effective tools for outreachand strengthening the university’s community engagement. Therefore, the objective of this work is to describe the process of creation and implementation of a Book Club in a Public Medical School.

## CASE PRESENTATION

The Escola Multicampi de Ciências Médicas do Rio Grande do Norte (EMCM), Federal University of Rio Grande do Norte (UFRN), was established in 2014 to address the urgent need to expand access to medical education in rural regions [12]. Its mission is to train physicians to work in underserved and rural areas, respecting the health, ethical, and cultural values of local communities. Approximately 280 students are currently enrolled in the medical program, distributed across all years of study.

Within this context, the Book Club was approved and funded by the Pro-Rectory of Extension, a department within the university responsible for managing and promoting community engagement and extension projects. The Book Club took place at the Medical School in a large auditorium with a capacity of about 50 people. Fourteen of the 22 Book Club participants were medical students, representing 63.6% of the total cohort and approximately 5% of the medical student body [13].

This research was classified by the project team as a public opinion study, with no possibility of identifying the participants, since data was collected anonymously and voluntarily. Therefore, in accordance with Article 2, item XIV of Resolution No. 510/2016 of the Brazilian National Health Council, submission to the CEP/Conep System (Research Ethics Committees and the National Research Ethics Commission - Conep) was not required [14].

### Formation of the Team

The Book Club team was formed with the primary objective of integrating literary discussions into the medical curriculum and promoting a dialogue between academia and the community. The selection of team members was based on their demonstrated interest in literature and willingness to contribute to the project. The team consisted of six first-year medical students, two professors, and one librarian. This team was responsible for planning and facilitating the activities for the 22 Book Club participants. The predominance of students reflects the Book Club's role as a space for extracurricular engagement and peer-led activities, while the presence of faculty and the librarian ensured academic rigor and intentional community outreach. The selection process was interest-driven rather than an institutional assignment, aiming to create a team passionate about humanistic approaches to health education.

In addition to the interdisciplinary structure of the team, the active involvement of the medical school librarian was crucial to the project success. Acting as deputy coordinator, the librarian contributed to the strategic planning of the Book Club and led efforts to build an accessible and inclusive literary collection, including direct contact with publishers to obtain books at reduced prices. Beyond these responsibilities, the librarian co-facilitated reading discussions and developed partnerships with librarians from other cities, fostering inter-institutional engagement. This role exemplifies how medical librarianship transcends administrative tasks, positioning the librarian as an agent of community outreach and humanistic education.

To ensure the cohesive functioning of the Book Club, tasks related to specific activities were distributed in pods, each with its respective representatives and assignments. The team planning meetings took place in person every fortnight to discuss the activities carried out by each pod, evaluate internal proposals and ongoing actions related to the Book Club’s implementation, and coordinate the upcoming meetings. The pods formed were coordination, organizational, communication and scientific dissemination, and products.

#### 1. Coordination Pod

This team was responsible for the development and submission of the initial Book Club project proposal to the Pro-Rectory of Extension of the University, for the development of the calendar of activities and the management of the other teams.

#### 2. Organizational Pod

This team carried out managing the space for the meeting’s reservation and organization, including the chairs arrangement, projection images, and participants’ reception.

#### 3. Communication and Scientific Dissemination Pod

The team conducted the necessary research for writing scientific papers and publications, academic materials, abstracts for congresses, and journalistic releases. This pod was created to ensure that the activities and outcomes of the Book Club could contribute to academic discourse and institutional visibility. The dissemination of the project’s results was included as a goal in the initial proposal, highlighting its potential as a model for similar educational and community engagement initiatives.

#### 4. Products Pod

This pod executed the production of the Book Club outreach materials, including posts and stories for Instagram (Meta). In addition, the team created the Book Club planner, records of the meetings, and maintains the Newsletter and the Book Club app.

### Selection of Works

To ensure equitable access to the selected books, the organizing team prioritized titles available in digital formats and negotiated with publishers to obtain complimentary or discounted copies. The university library supported this initiative by incorporating some of the selected works into its collection. No participant was required to purchase a book to participate in the discussions, ensuring that financial limitations would not be a barrier to engagement.

The works selected for the Book Club were chosen through a process of research and consultation with experts in the fields of sociology, anthropology, and education. Initially, fifteen book options were compiled which addressed essential and relevant topics for public and academic debate, including racism, gender, violence, grief, and colonialism. This resulted in the final selection of six works (Table 1). The selection of the final six books was carried out based on criteria such as attractiveness for the Book Club’s target audience, critical reception, and text length—favoring those under 300 pages. Special attention was given to the accessibility of the books, including the availability of digital formats and affordable pricing (ideally under R$50 or US$9.16), to facilitate acquisition by the library and to support broader availability for those who wished to purchase their own copy. The list of readings for the 2023 Book Club can be found in Table 1.

**Table 1:** Selected works for the 2023 Book Club.

Book	Author	Thematic
“The Dark Side of Skin”^(15)^	Jeferson Tenorio	Racism; police violence
“Happening”^(16)^	Annie Ernaux	Abortion; law and its imperative on female bodies
“The Year of Magical Thinking”^(17)^	Joan Didion	Death; grief
“Crooked Plow”^(18)^	Itamar Vieira Junior	Violence against women; enduring slave practices; poverty; violence; racism
“In the Eye of the Wild”^(19)^	Nastassja Martin	Complexity of life; construction of empowering narratives
“The Sound of the Jaguar's Roar”^(20)^	Micheliny Verunschk	Indigenous peoples; civilizational theory

These literary works stand out for the depth with which they address fundamental themes of the human experience, especially regarding racism, gender, grief, trauma and colonialism. By exploring the lives of Black people in Brazil, the books “The Dark Side of Skin” by Jeferson Tenório [15] and “Crooked Plow” by Itamar Vieira Junior [18], encourage a more empathic and critical understanding of the racial issues that permeate Brazilian society. Annie Ernaux's “Happening” [16] and Joan Didion's “The Year of Magical Thinking” [17] offer deeper perspectives on the female body and grief, respectively, stimulating recognition of the importance of dignity, autonomy and individuality in the processes of illness and healing. Nastassja Martin's [19] narrative in “In the Eye of the Wild” underscores the power of personal history and resilience, showing how emotional and psychological context is an integral part of a person's treatment and recovery. Finally, Micheliny Verunschk's “The Sound of the Jaguar's Roar” [20] confronts participants with the history of colonial violence, encouraging reflection on the historical and cultural impacts on the health of Indigenous populations.

### Implementation of the Book Club: start of activities

Once the planning phase was completed, the Book Club began its activities and expanded the diversification of participants with the Book Club's dissemination strategies.

### Social media: Instagram (Meta)

**Figure 1:**
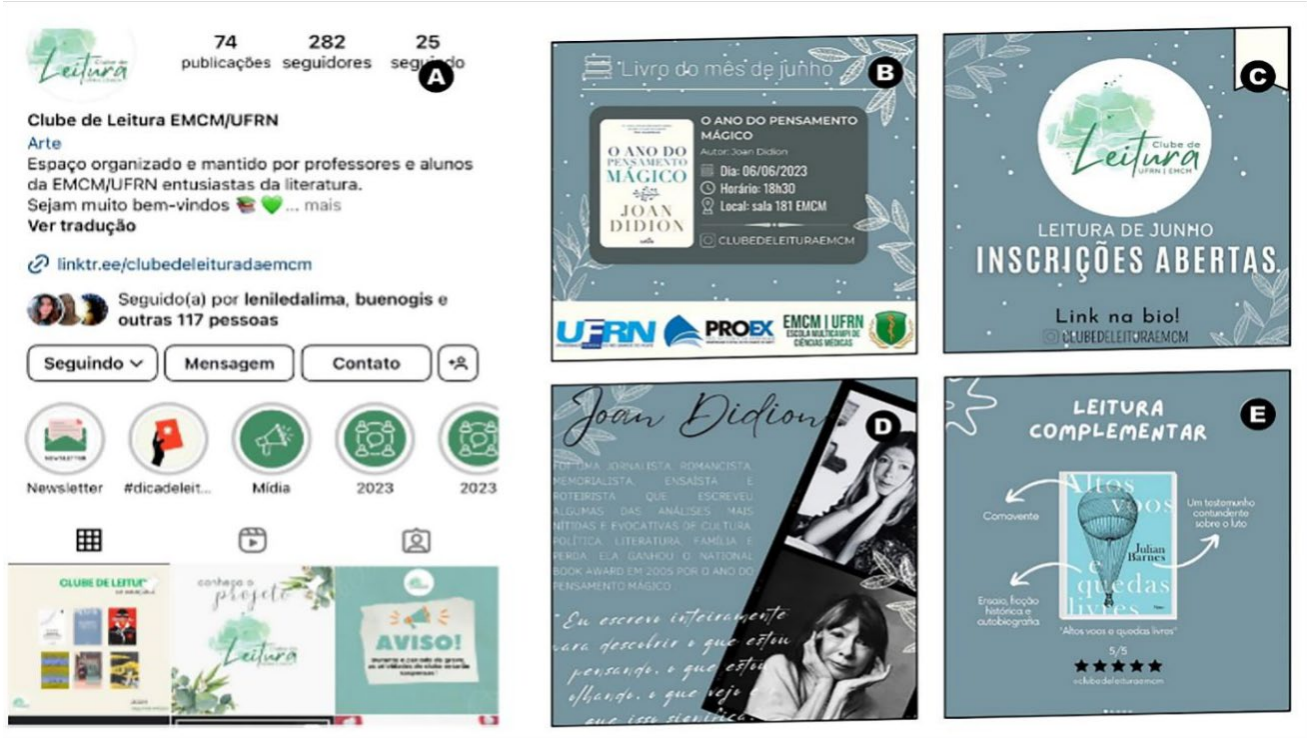
Communications of the meetings for all communities. A) Book Club page on Instagram (META). B) Post with the data (date, time and place). C) Publication of the opening of registrations. D) Post a mini-biography about the author of the book. E) Post with an indication of complementary readings to the theme of the month.

The public announcement of the Book Club after the completion of the planning stage was crucial to start activities. For this reason, publicly, the beginning of the Book Club was marked by the creation of a profile on the social network Instagram (Meta), a platform widely used by the general public (Figure 1A). This network was a key tool for sharing content and reaching the target audience. Instagram (Meta) plays a key role in the dissemination of the Book Club, both to the internal community of the college and to the external public[21].

**Figure 2:**
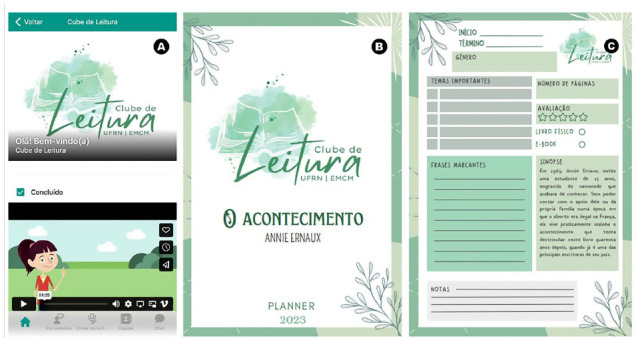
Mobile application. A) Home page of the Book Club App, in which the project is presented by the fictional character Ana Karenina. B) Planner cover. C) Contents of the club planner for reading the book “Happening” (1991).

Posts on Instagram (Meta) began a month before each meeting so that participants had time to read the selected work. The first post included detailed information about the meeting: location, date and time, as well as the title of the chosen work (Figure 1B). Then, the opening of the inscriptions was disclosed (Figure 1C). Subsequently, two new publications presented a mini-biography of the author (Figure 1D), and indications of complementary readings to the theme addressed by the book of the month (Figure 2E). In this way, we sought to encourage interest in the universe of works selected from constant and strategic information.

### Registration

Book Club registration was conducted through the University’s system and remained open for four days following the public announcement. After the end of the registration period, the club staff sent an email to all registrants to confirm the date, time and place of the meeting. In addition, the email provided a link to access the Book Club app and the reading planner of the month, developed exclusively for participants.

### Club App and Planner Notebook

The app for the Book Club was developed in the Glide Apps^®^. The app provides several features, including a brief account of the history of the Book Club, presented by a fictional character named Anna Karenina (Figure 2A). Participants also had access to the list of books of the year, available for consultation in the virtual environment. In addition, the app included an interactive chat, allowing for the exchange of information between participants and Book Club staff. In this space, it was possible to discuss the readings, to ask questions related to the meetings and share recommendations for literary and non-literary content relevant to the topics addressed. In addition, a planner was available to help participants organise their reading schedule, reflect on key themes, and track their progress through the selected books (Figures 2B). This planner contained spaces for listing important themes addressed by the work, recording striking phrases and a space for the reader to take notes freely (Figures 2C).

### Book Club Meetings

The Book Club meetings were held monthly, resulting in 6 meetings, in which each month a literary work selected by the team would be discussed. The average duration of the meetings was 90 minutes and each concluded with a brief period of informal socialization among participants. During the meetings, a slide was projected, featuring thematic elements such as color palettes, quotes, and highlights designed to evoke the atmosphere and core themes of the book under discussion. Besides, discussion circles promote open communication, where all participants feel equally valued and encouraged to share their perspectives [22]. The arrangement of the chairs in a circle during the meetings was chosen as a pedagogical strategy, since it contributed to the University and community working together, in contrast to the ineffective tradition of the first acting on the second unilaterally and imposing their beliefs and opinions [23].

The meetings followed a structured sequence of activities. Initially, an introduction to the work was presented, which included a summary, the political and social context at the time of publication, and a brief biography of the author. This stage had an average duration of 10 minutes. Then, the discussion was opened to the participants, who shared their opinions, ideas and personal experiences, enriching the group with multiple perspectives on the same work. The discussion lasted approximately 1 hour. Finally, the last 20 minutes were dedicated to a brief period of informal socialization among participants, often accompanied by a small snack.

## DISCUSSION

Reading clubs are a pedagogical initiative to integrate not only the humanities into the health curriculum, but also to create closer dialogue between the University and community. As in other regions of Brazil, the region of Sertão, needs this kind of dialogue between future doctors and the local community to foster mutual understanding, promote empathy, and encourage more effective communication. This exchange helps medical students to better understand the cultural, social, and economic realities of the populations they will serve, while also empowering community members to actively participate in health-related discussions. In this work, we aim to describe the process of creating and implementing a reading club in a medical school in the Brazilian Sertão.

In 2023, the book club received 46 applications, with 22 participants, of whom 64% were medical students and 36% were members of the community. The participation of medical students reflects their interest in extracurricular activities that complement academic training. For students, this highlights the value of initiatives that go beyond clinical training to support interpersonal skills and personal growth, contributing to a more well-rounded and humanistic medical education [9].

For the community, the meetings at the college itself were a plan of rapprochement between academia and the local population, making the institution feel more accessible and welcoming [24]. By eliminating perceptions of hierarchy, the environment becomes inclusive, facilitating an open and constructive dialogue among participants [25]. However, given the low community participation, it is necessary to implement new strategies, such as increasing financial resources for Federal University extension initiatives and including the community in the decision-making process of the projects in which they are involved. Although no formal target was established, the team had anticipated greater interest from the local population, aiming for more balanced participation between academic members and the broader community. This challenge is consistent with previous observations in the literature, which highlight the barriers that socially excluded communities often face in engaging with higher education initiatives. [26].

Before the face-to-face meeting, the participants had access to the chat, the reading planner and the social networks of the Book Club. In the interactive chat on the app, the participants shared recommendations for literary and non-literary content relevant to the topics covered. Something similar happened on the Book Club’s social media; however, in this case, the content was publicly accessible to both participants and non-participants. The use of digital resources promotes the integration and engagement of members, facilitating access to a variety of materials and enriching discussions with diverse perspectives [27]. These resources allow for more agile and dynamic communication, potentially expanding the reach and influence of the Book Club. The reading planner, on the other hand, assisted participants in managing their time and monitoring their progress in reading, providing a more structured and engaging experience [28].

The network of readers created through the Book Club’s Instagram (Meta) profile showed significant interest from the local population. Metrics from the page revealed that out of 282 followers, the majority were residents of the surrounding region, indicating strong community engagement. This suggests that the club’s themes resonate locally and that the initiative may serve as an effective educational strategy to promote reading in a region where only 48% of the population reports having read at least one book in the past three months — a historical challenge in the Northeast of Brazil. [29].

In addition, throughout the implementation of the Book Club, it was observed that most participants belonged to the 25–34 age group. This contrasts with findings from the 2019 Pro-Book Institute (IPL)[29] survey, which identified children and adolescents (11–17 years old) as the most active readers in the country. This discrepancy may stem from several factors, such as the thematic choices and language used in the Book Club, which might not yet fully attract a younger audience.

In our Book Club we had more adults present than adolescents; however, their engagement was still limited by competing responsibilities and time constraints, which are common among individuals aged 18 to 60 [30]. Therefore, it is important to continue diversifying the selected works and formats to ensure accessibility and appeal across different age groups within the community.

Besides, while the Book Club’s primary audience was university students and adult community members, there had been hope that some younger readers might also join. The absence of adolescents may be related to the complexity of the selected books, which addressed mature themes such as grief, racial violence, and colonial trauma. In addition, the Book Club did not implement marketing strategies specifically aimed at youth, nor does the medical school currently have an outreach program targeting adolescents. Future iterations may consider including youth-friendly genres, such as fantasy or young adult literature, and forming partnerships with local schools to encourage broader participation.

In relation to gender, it was noted that women showed greater interest and engagement in the Book Club activities. This trend aligns with the findings of the IPL survey [29], which highlights a stronger participation of women in reading practices. Historically, this pattern can be linked to the limited access women had to public life and the labor market, which made reading one of the most accessible cultural and leisure activities [31]. Despite social advances, women continue to be more engaged in literary initiatives. The selection of books by the club, which often addressed themes related to female experiences and perspectives, may have contributed to this strong female participation. At the same time, these themes encouraged meaningful contributions from male participants, who engaged in the discussions from different viewpoints, thereby enriching the dialogue with diverse gender perspectives.

Although no formal survey was conducted in the first iteration of the Book Club, anecdotal feedback from participants highlighted the importance of the meetings as safe spaces for expression and reflection. Some participants described the discussions as “therapeutic” and “essential for seeing the world beyond the hospital walls.” These informal accounts reinforced the role of the Book Club as a supportive environment for developing humanistic skills.

The Book Club project proved to be a powerful strategy for integrating the academic and external community, strengthening bonds and fostering critical discussions through literature. The experience of in-person meetings revealed the transformative potential of open dialogue between students and community members, highlighting reading as a tool for empathy, active listening, and civic development. Since its implementation, the Book Club has evolved and expanded beyond its initially intended audience. The Book Club continued beyond its initial cycle in 2023, expanding to new audiences, such as elderly community members and children in public schools. While data from subsequent cycles is still being collected and compiled for future analysis, preliminary observations suggest a broader demographic reach and growing engagement. A comparative study is planned to assess the evolution of participation and thematic focus over time.

What makes this Book Club project unique is its implementation within a public medical school located in a rural region of Brazil, designed not only to humanize medical education but also to create a sustained bridge between academia and the surrounding community through literature. The specific goals of the project were to promote critical reflection, encourage empathy and communication among medical students, and strengthen the university's social engagement. These goals were achieved, as evidenced by the active participation of students and the university extension project to broader audiences, including the elderly and children in later editions.

For those interested in developing similar initiatives, we recommend building a multidisciplinary team with diverse skills and fostering strong institutional support from university departments and libraries. It is important to consider local context, accessibility of materials, and inclusive outreach strategies. The contribution of each participant was vital: students brought enthusiasm and creativity; professors provided academic rigor and mentoring; and the librarian offered expertise in content curation, logistics, and inter-institutional partnerships, ensuring equitable access to books and acting as a cultural mediator. This synergy demonstrated how interdisciplinary collaboration can transform reading into an educational experience that also fosters social engagement and emotional connection.

## Data Availability

There is no data associated with this article.

## References

[1] Mega MN, Bueno BC, Menegaço EC, Guilhen MP, Pio DAM, Vernasque JRS. Experiência de estudantes com a literatura na formação médica. Rev Bras Educ Med. 2021;45(2):e20200226. DOI: 10.1590/1981-5271v45.2-20200226

[2] Brasil. Resolução CNE/CES nº 3, de 20 de junho de 2014. Diário Oficial da União, Seção 1. 2014 Jun 20;8-11.

[3] Smydra R, May M, Taranikanti V, Mi M. Integration of arts and humanities in medical education: A narrative review. J Cancer Educ. 2021;37(5):1267–1274. DOI:10.1007/s13187-021-02058-3. 34319566

[4] Garcia Júnior CAS. Humanidades: Ensino de “nova” dimensão ética na educação médica. Rev Bioet. 2020;28(3):479–485. DOI:10.1590/1983-80422020283410.

[5] Mangione S, Chakraborti C, Staltari G, Harrison R, Tunkel AR, Liou KT, et al. Medical students’ exposure to the humanities correlates with positive personal qualities and reduced burnout: A multi-institutional U.S. survey. J Gen Intern Med. 2018;33(5):628–634. DOI:10.1007/s11606-017-4275-8. 29380213 PMC5910341

[6] Barbosa-Medeiros M, Caldeira AP. Saúde mental de acadêmicos de medicina: estudo longitudinal. Rev Bras Educ Med. 2021;45(3):e20190285. DOI:10.1590/1981-5271v45.3-20190285.

[7] Rios IC. Humanidades e medicina: razão e sensibilidade na formação médica. Cien Saude Colet. 2010;15(1):1725–1732. DOI:10.1590/s1413-81232010000700084. 20640334

[8] Harlow T. “Profound courtesy”: literature and poetry in medicine. Lit Med. 2020;38(2):282–300. DOI:10.1353/lm.2020.0022 33518544

[9] Ney DB, Ankam N, Wilson A, Spandorfer J. The implementation of a required book club for medical students and faculty. Med Educ Online. 2023;28(1):2173045. DOI:10.1080/10872981.2023.2173045 36718544 PMC9891158

[10] Groner LK, Lee M, Gil HDJ, Min RJ, Babagbemi K. Hiding in plain sight: how incorporating honest discussion of racial and social (in)justice into medical education can inspire change. Clin Imaging. 2022;89:37–42. DOI:10.1016/j.clinimag.2022.04.018 35696946

[11] Valente TA, Domingos JR. Clube de leitura: estratégia para formação de leitores. Rev Leia Escola. 2019;19(3):[sem paginação].

[12] Oliveira ALO, Melo LP, Pinto TR, Azevedo GD, Santos M, Câmara RBG, et al. Vivência integrada na comunidade: inserção longitudinal no sistema de saúde como estratégia de formação médica. Interface (Botucatu). 2017;21(1):1355–1366. DOI:10.1590/1807-57622016.0533.

[13] Escola Multicampi de Ciências Médicas. [Internet]. 2024. Available from: https://www.emcm.ufrn.br/.

[14] Brasil. Conselho Nacional de Saúde. Resolução nº 510, de 7 de abril de 2016. Dispõe sobre as normas aplicáveis a pesquisas em Ciências Humanas e Sociais. Diário Oficial da União. 2016 maio 24;Seção 1:44.

[15] Campos DP. RioGrandedoNorte Municip Caico.svg [Internet]. 2006. Available from: https://pt.m.wikipedia.org/wiki/Ficheiro:RioGrandedoNorte_Municip_Caico.svg.

[16] Tenório J. O avesso da pele. São Paulo: Companhia das Letras; 2020.

[17] Ernaux A. O acontecimento. São Paulo: Fósforo Editora; 2022.

[18] Didion J. O ano do pensamento mágico. Rio de Janeiro: Nova Fronteira; 2006.

[19] Vieira Junior I. Torto arado. São Paulo: Todavia; 2019.

[20] Martin N. Escute as feras. São Paulo: Editora 34; 2021.

[21] Verunschk M. O som do rugido da onça. São Paulo: Companhia das Letras; 2021.

[22] Morais NSD, Brito MLA. Marketing digital através da ferramenta Instagram. E-Acadêmica. 2020;1(1):e5. Available from: https://www.eacademica.org/eacademica/article/view/5.

[23] Scliar M. A face oculta: inusitadas e reveladoras histórias da medicina. Porto Alegre: Artes e Ofícios; 2001.

[24] Fernandes MC, Silva LMS, Machado ALG, Moreira TMM. Universidade e a extensão universitária: a visão dos moradores das comunidades circunvizinhas. Educ Rev. 2012;28(4):169–194. DOI: 10.1590/S0102-46982012000400007

[25] Benneworth P, editor. University engagement with socially excluded communities. Springer; 2013.

[26] Veroneze CC, Javarez JG, Nadal LMK. Clubes de leitura em movimento: integração nas bibliotecas do IFPR. Rev Bras Bibliotecon Doc. 2019;15:314–326. Available from: http://hdl.handle.net/20.500.11959/brapci/127492.

[27] Baltazar CC, Fernandes T. Plataformas digitais como incentivo e promoção da leitura: um estudo de caso sobre a Tag - experiências literárias. In: Anais do 17º Encontro Internacional de Arte e Tecnologia; 2018; Brasília, DF. Brasília (DF): [publicadora]; 2018. p. 71-82.

[28] Pereira AS, Santos RLL. Como um livro sendo relido: relato de experiência sobre o clube PET de leitura. Rev Areia. 2021;4(5):48–57.

[29] Instituto Pró-Livro. Retratos da leitura no Brasil. 5ª ed. [Internet]. 2019. Available from: https://www.prolivro.org.br/5a-edicao-de-retratos-da-leitura-no-brasil-2/a-pesquisa-5a-edicao/.

[30] Carniel M. Poesia e jovem leitor: possíveis intervenções pedagógicas [monografia]. Caxias do Sul: Universidade de Caxias do Sul; 2020.

[31] Brito ACC. Literatura de entretenimento como convite: para além da formação de leitoras [monografia]. Brasília: Universidade de Brasília; 2021.

